# Finite element analysis of the knee joint stress after partial meniscectomy for meniscus horizontal cleavage tears

**DOI:** 10.1186/s12891-023-06868-y

**Published:** 2023-09-19

**Authors:** Hao Chen, Lantao Liu, Youlei Zhang

**Affiliations:** 1 Department of Sport Medicine, Beijing DCN Orthopedic Hospital, No.19 Fushi Road, Beijing, 100143 China; 2 Department of Spinal Medicine, Qingdao Hospital, University of Health and Rehabilitation Sciences, Qingdao Municipal Hospital, No.5 Donghai Zhong Road, Qingdao, 266000 China

**Keywords:** Meniscus, The knee joint, Finite element simulation

## Abstract

**Objective:**

To establish a finite element model of meniscus horizontal cleavage and partial resection, to simulate the mechanical changes of knee joint under 4 flexion angles, and to explore what is the optimal surgical plan.

**Methods:**

We used Mimics Research, Geomagic Wrap, and SolidWorks computer software to reconstruct the 3D model of the knee joint, and then produced the horizontal cleavage tears model of the internal and lateral meniscus, the suture model, and the partial meniscectomy model. These models were assembled into a complete knee joint in SolidWorks software, and corresponding loads and boundary constraints were added to these models in ANSYS software to simulate the changing trend of pressure and shear force on femoral condylar cartilage, meniscus, and tibial cartilage under the flexion angles of 0°, 10°, 20°, 30° and 40° of the knee joint. At the same time, the difference of force area between medial interventricular and lateral interventricular of knee joint under four states of bending the knee was compared, to explore the different effects of different surgical methods on knee joint after horizontal meniscus tear.

**Results:**

Within the four medial meniscus injury models, the lowest peak internal pressure and shear force of the knee joint was observed in the meniscal suture model; the highest values were found in the bilateral leaflet resection model and the inferior leaflet resection model; the changes of pressure, shear force and stress area in the superior leaflet resection model were the most similar to the changes of the knee model with the meniscal suture model.

**Conclusion:**

Suture repair is the best way to maintain the force relationship in the knee joint. However, resection of the superior leaflet of the meniscus is also a reliable choice when suture repair is difficult.

## Introduction

The main function of the meniscus is to transfer and distribute gravitational loads over a large area of articular cartilage, which has the function of stabilizing the joint and absorbing vibrations during knee motion [1, 2]. Horizontal cleavage tears (HCT) are one of the most common meniscal injuries, the tear extends inward from the level of the free edge of the meniscus, dividing the meniscus into two layers, the superior and inferior [3]. HCT is often treated by arthroscopic partial meniscectomy (hereafter referred to as APM) instead of repair [4]. The main reason is that HCT usually occurs in the “white-white” zone of the meniscus, i.e., the avascular zone, and the lack of blood flow in this area greatly affects meniscal healing, and even if the meniscus is repaired by suturing, the prognosis is not satisfactory [5]. To date, numerous studies have found that the incidence of osteoarthritis of the knee is greatly increased after meniscectomy [6, 7], because the mechanical balance within the knee joint is altered after meniscectomy, leading to early onset of joint wear and degeneration [8]. Currently, the treatment of HCT by APM includes three main forms of resection: superior leaflet, inferior leaflet, and bilateral leaflet, but we do not yet know the biomechanical effects on the knee joint after these procedures. Beamer [3] and Koh [9] performed mechanical analysis by simulating meniscal laminar fractures in a cadaveric model and found that different resection methods had different effects on the mechanical relationships within the knee joint.

Some trials have confirmed an approximately 50% reduction in the tibiofemoral contact area and a 2–3-fold increase in contact force after partial meniscectomy [8, 10], and also found that the peak shear force decreased with increasing contact area after unilobar or bilobar resection of the HCT, reaching levels similar to other meniscectomies [7, 11, 12]. However, previous cadaveric studies have obtained HCT models by surgically destroying the structures of the meniscus, and the process of surgically exposing the meniscus itself destroys the stable structures of the knee joint, such as the lateral collateral ligaments of the knee joint [3, 9]. The finite element technique can establish the lower limb bone and muscle model by computer, and then add corresponding load and boundary constraint to carry out mechanical analysis [13, 14]. Although there are many reports on the mechanical analysis of knee joint finite element models, there are few studies on the modeling of HCT [15–17]. In this study, we simulated the effects of horizontal meniscal tear and surgical resection on the knee joint for mechanical simulation analysis and performed a complete analysis of knee joint mechanics after meniscal injury and partial meniscectomy. In addition, all components in our knee joint model (including bone, cartilage, and ligament) are derived from the same MR data, ensuring that all components share the same coordinate system. This method is different from the method of extracting bone model and soft tissue model from CT data and MRI data respectively, and then manually assembling them in SW software. It can effectively avoid the error caused by an inconsistent coordinate system.

## Material and methods

### Data acquisition

We selected a 31-year-old healthy male volunteer with a height of 175 cm and a weight of 70 kg. The right knee joint was scanned by a 1.5T MRI machine (ESSENZA, Siemens) from sagittal, coronal and transverse directions, and a T2 proton-weighted image was obtained with a layer thickness of 1.5 mm, spacing of 0 mm, a matrix of 192 × 320, a field of view size180mm. The MRI image data were saved as DICOM files. All DICOM files were imported into Mimics Research 21.0 software (Materialise, Belgium) for the initial 3D reconstruction of each structure of the knee joint. Nineteen solid structures including bone, cartilage, ligament, and meniscus were created and saved as STL format files.

### Reconstruction of the 3D model of the knee joint

**Fig. 1:**
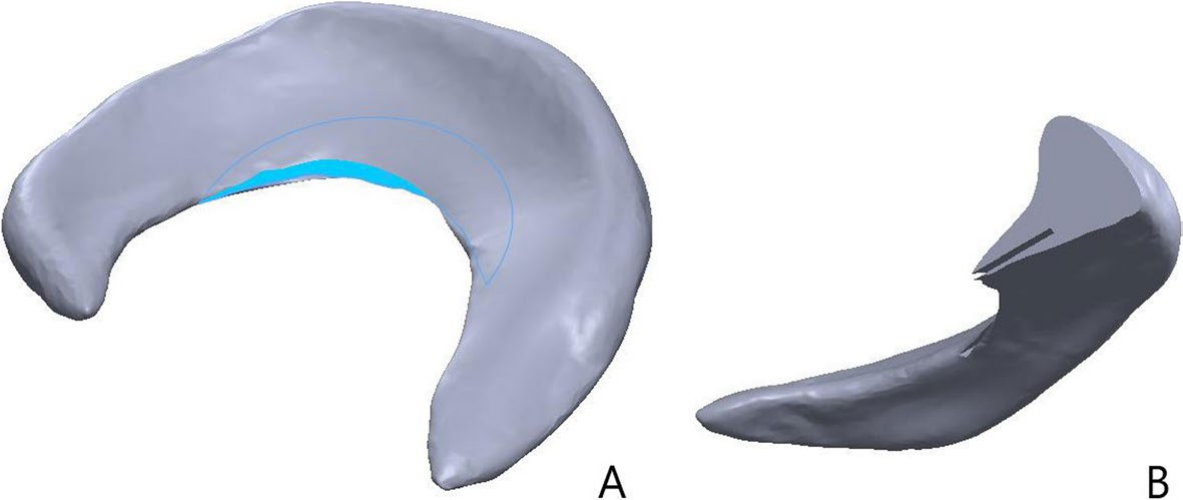
Three-dimensional model of the HTC. **A** The blue arc area is the horizontal tearing range. **B** The meniscus profile shows a horizontal tear deep into the meniscus body

We imported the STL files into Geomagic Wrap 2017 software (Geomagic Corp., USA) to perform surface smoothing and generate surface slices, and saved them as STEP files. All STEP files were imported into SolidWorks 2020 software (Dassault, France) for part assembly and interference elimination, and the “stretch-excision” command was used to simulate the horizontal meniscal tear and partial meniscal resection after surgery (Fig. 1). The lateral meniscus model had a total unilateral area of about 642.91 mm^2^ and a volume of about 1430.65 mm^3^. The horizontal tear of the lateral meniscus was located in the body of the meniscus, and the horizontal tear covered an area of about 73.63 mm^2^, accounting for about 11.45% of the total unilateral area, and the resection volume of the superior leaflet was about 72.39 mm^3^, accounting for about 5.06% of the total volume. The volume of inferior leaflet resection is about 63.25mm^3^, accounting for about 4.42% of the total volume, the volume of bilobar resection is 135.64mm^3^, accounting for about 9.48% of the total volume; the total unilateral area of medial meniscus is about 830.16 mm^2^, the volume is about 2389.29 mm^3^, the horizontal fissure of medial meniscus is located in the body of meniscus, the area covered by horizontal fissure is about 86.66mm^2^, accounting for about 10.44% of the total unilateral area. The volume of superior leaflet resection was about 69.86 mm^3^, accounting for about 2.93% of the total volume, the volume of inferior leaflet resection was about 138.39 mm^3^, accounting for about 5.79% of the total volume, and the volume of double leaflet resection was 207.65 mm^3^, accounting for about 8.69% of the total volume.

**Fig. 2:**
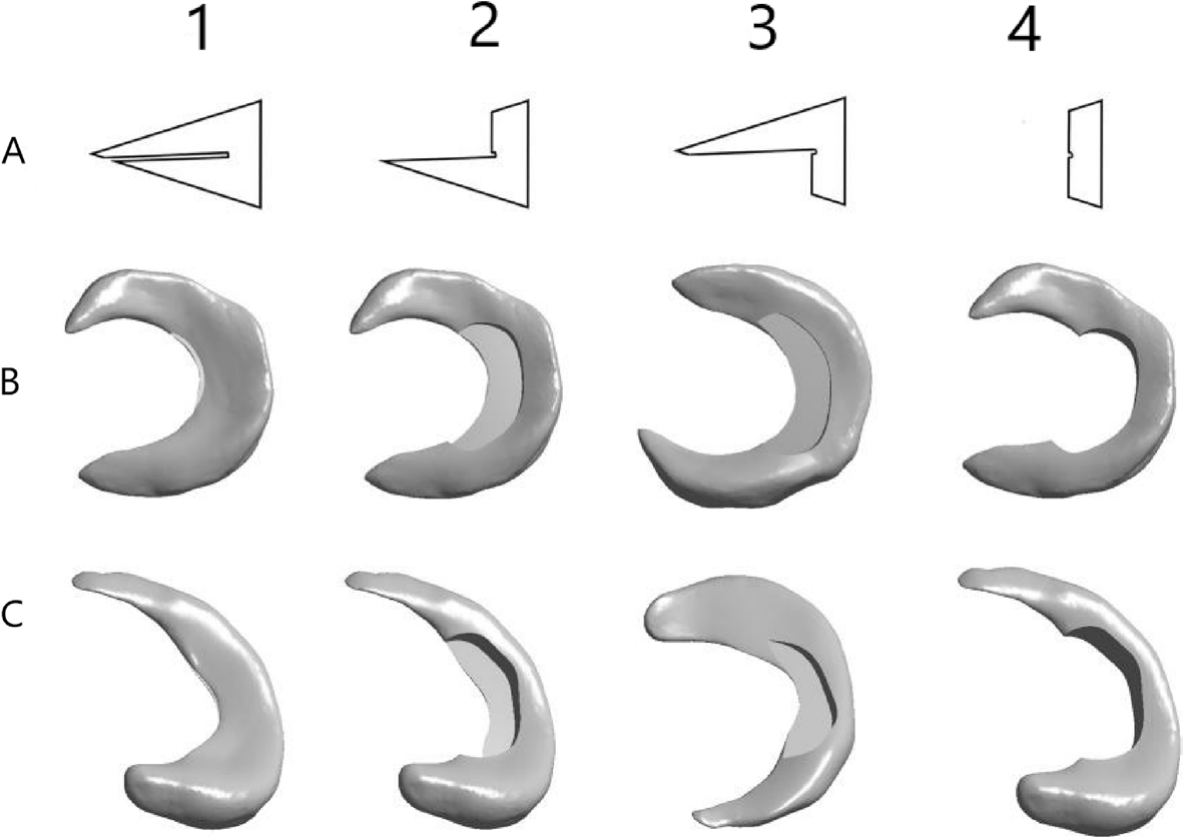
The Three-dimensional model view of the meniscus. **A** Sketch of partial meniscus resection. **B** Three-dimensional model of medial meniscus. **C** Three-dimensional model of lateral meniscus. 1: Three-dimensional model of the keen meniscus Horizontal Cleavage Tears. 2: Three-dimensional model of partial meniscectomy (superior part). 3: Three-dimensional model of partial meniscectomy (inferior part). 4: Three-dimensional model of partial meniscectomy (both superior and inferior part)

**Fig. 3:**
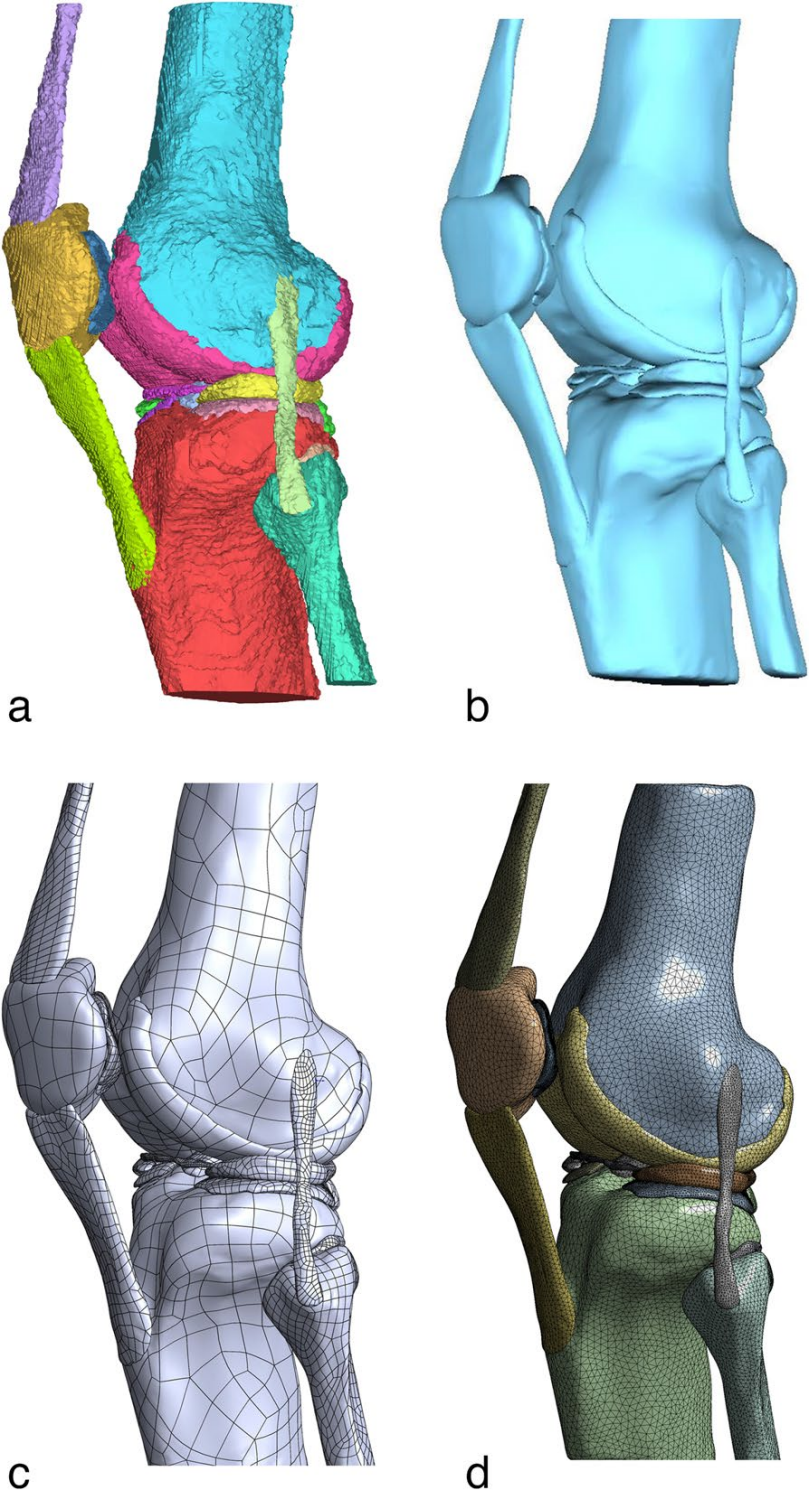
3D model for mechanical analysis in ANASYS software. **A** Primary model generated in mimics. **B** Primary model in Geomagic Wrap for surface smoothing. **C** Interference elimination between the parts in solidwork. **D** Meshing in anasys

The 3D models of the knee joint were finally generated, consisting of 4 bones, 5 cartilages, 8 ligaments, and 8 menisci including four medial meniscus models and four lateral meniscus models. Each component shares a coordinate system, and we assembled all the pieces into a complete knee joint by “overlapping the origin point” in Solidworks (Figs. 2 and 3).

After the above steps, we imported eight 3D models of the knee joint into ANASYS 18.0 software (ANASYS Corp., USA) and reconstructed different knee structures using tetrahedral cells with 2.0 mm, 1.5 mm, 1.0 mm, and 0.8 mm mesh sizes, respectively. The mesh size of each component is shown in Table 1. The model was further optimized using this method to divide the entire knee joint into 1,087,763 nodes and 729,505 elements (Fig. 3).

**Table 1:** Mesh size of each component

Mash size (mm)	Component
2.0	Bone (femoral, tibial, patella, fibula)
1.5	Ligament (patellar ligament, quadriceps tendon, anterior cruciate ligament, posterior cruciate ligament)
1.0	Meniscus, medial and lateral collateral ligaments, plate-femoral ligament, transverse knee ligament
0.8	Articular cartilage (femoral cartilage, tibial cartilage, fibula cartilage,)

### Knee joint model parameter settings

We refer to the material parameters from previous finite element studies to assign values to each part of the 3D model, and due to the small deformation of the bone structure in mechanical experiments, we assume that it is a rigid material [18] and the articular cartilage is an isotropic linear elastic material [19]. Due to the large deformation and anisotropy of the cruciate ligament structure, we used the Neo-Hookean hyperelastic model to model the ductility of the ACL and PCL [17], and the other ligaments as isotropic linear elastic materials [19]. The meniscus is composed of water, collagen, and proteoglycans, and the collagen matrix within the meniscus provides cyclic stress to resist shear forces in the joint compartment and prevent the meniscus from expanding outward. When the joint is loaded, this property causes the meniscus to deform less horizontally than longitudinally [20, 21]. Based on this, we considered the meniscus as a transversely isotropic material and set different elastic moduli in the circumferential, axial, and radial directions after the model was established in a columnar coordinate system. All engineering data are shown in Table 2.

**Table 2:** Engineering parameter setting of each part of the knee joint 3D model

	Material Properties	Modulus of elasticity (MPa)	Poisson’s ratio	Shear modulus C1	Non-shear shrinkage parameter D1
Bone	Rigidity	-	-	-	-
Articular cartilage	Isotropic linear elasticity	15	0.3	-	-
Anterior Cruciate Ligament	Superelastic	-	-	5.08	0.00683
Posterior cruciate ligament	Super-elastic	-	-	6.06	0.0041
Medial and lateral collateral ligaments	Isotropic linear elasticity	60	0.3	-	-
Patellar ligament/quadriceps tendon	Isotropic linear elasticity	225	0.3	-	-
Plate-femoral ligament	Isotropic linear elasticity	60	0.3	-	-
Transverse knee ligament	Isotropic linear elasticity	60	0.3	-	-
meniscus	Cross-sectional isotropic	Circumferential 120	0.4	-	-
		Radial 20	0.4	-	-
		Axial 20	0.4	-	-

### Boundary constraints and loads

#### Boundary constraints

The contact surfaces between bone and cartilage and bone and ligament were set to be bound, the tibial plateau was fixed between the anterior and posterior horn of the medial and lateral meniscus, the meniscus was sliding between the femoral cartilage and the patellofemoral joint surface, and the friction coefficient was 0.014 [22], the friction coefficient between the cartilage and the meniscus was set to 0.06, and the friction coefficient between the HCT resection area and the cartilage contact surface was set to 0.09 [23]. In this experiment, we set the distal femur and distal tibia to be fully fixed, set the femur to rotate counterclockwise along the femoral through-condylar line [24], simulated knee flexion, and limited all degrees of freedom except for that.

#### Model verification

It is well known that Anterior Drawer Test (ADT) and Pivot Shift Test (PST) are important ways to evaluate the stability of the knee joint [25]. When the bending of the original knee joint model was 0 degrees, 134N posterior femur load was added to the midpoint of the femoral condyle line to simulate the ADT. A 10Nm valgus torque and a 5Nm internal rotation torque were applied to the knee joint to simulate the axial displacement test. The displacement results and rotation Angle obtained were similar to the cadaver experimental data of Gabriel [26] and the finite element simulation experimental data of Song [27], these data can prove the validity of this model. As shown in Table 3.

**Table 3:** Validation parameters of knee joint model

	Flexion angle (°)	Anterior–posterior translation (mm)	Proximal–distal translation (mm)	Medial–lateral translation (mm)	Valgus rotation (°)	Internal rotation (°)
ADT	0	4.5	0.4	1.4	0.6	2.9
	15	6.4	0.7	2.5	1.4	4.5
	30	7.2	1.2	3.3	3.6	8.1
PST	15	5.5	0.3	2.9	4.5	21.4
	30	7.9	0.9	1.6	7.2	26.6

#### Load and knee flexion angle selection

**Fig. 4:**
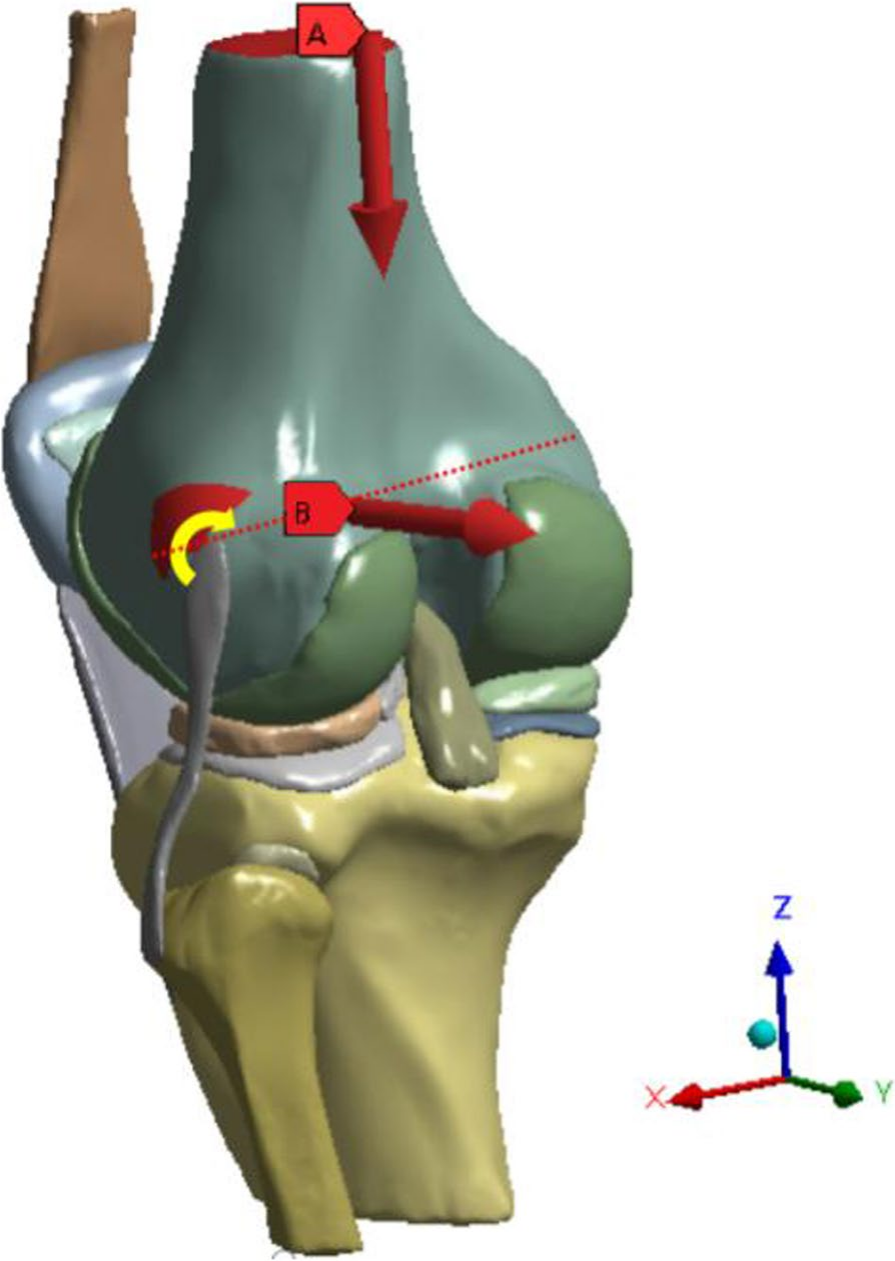
Schematic diagram of loads. **A** Axial load perpendicular to the horizontal plane. **B** Horizontal displacement load of the femur posteriorly. The red dotted line is the passondylar line, and the femur is set to rotate counterclockwise with the passondylar line as the axis

**Fig. 5:**
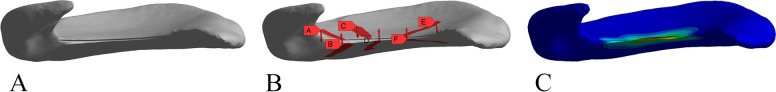
Simulation process of meniscus suture. **A** Horizontal meniscus tear model. **B** Three sets of reversal forces are applied to the upper and lower part of the meniscus in the area of horizontal meniscus fracture. **C** Displacement cloud showing that the meniscus has closed in the area of force

Since the knee movement simulated by ASTM F3141-15 is more similar to human gait [28], we converted the 100 kg data to 70 kg data in this study by referring to the ASTM F3141-15 standard. Based on the complete gait cycle data, we believe that the frequent changes of small-angle knee flexion become the main factor aggravating knee degeneration, so we selected five knee flexion angles of 0°, 10°, 20°, 30°, and 40°, and applied axial load and horizontal displacement load to the femur. Since the ASTM F3141-15 standard lacks data for 10°, 20°, 30°, and 40° of knee flexion, we took three sets of approximate values for converting axial loads: 0° (700N), 10.31° (612.59N), 20.1° (1442.87N), 29.33° (900.98N), 39.12° (498.73N) [29]. Also, based on the results of Peña AE [30] and Halonen KS [31], we added a posterior load of 134 N at the midpoint of the line connecting the midpoints of the femoral inner and outer condyles along the vertical coronal plane (Fig. 4). In the HCT model, we applied three sets of forces in opposite directions to the horizontal meniscal fracture region to achieve a simulated state of the meniscus after suturing (Fig. 5).

## Results

### Analysis of internal pressure in different angles of knee joint flexion

#### Medial meniscus injury model

**Fig. 6:**
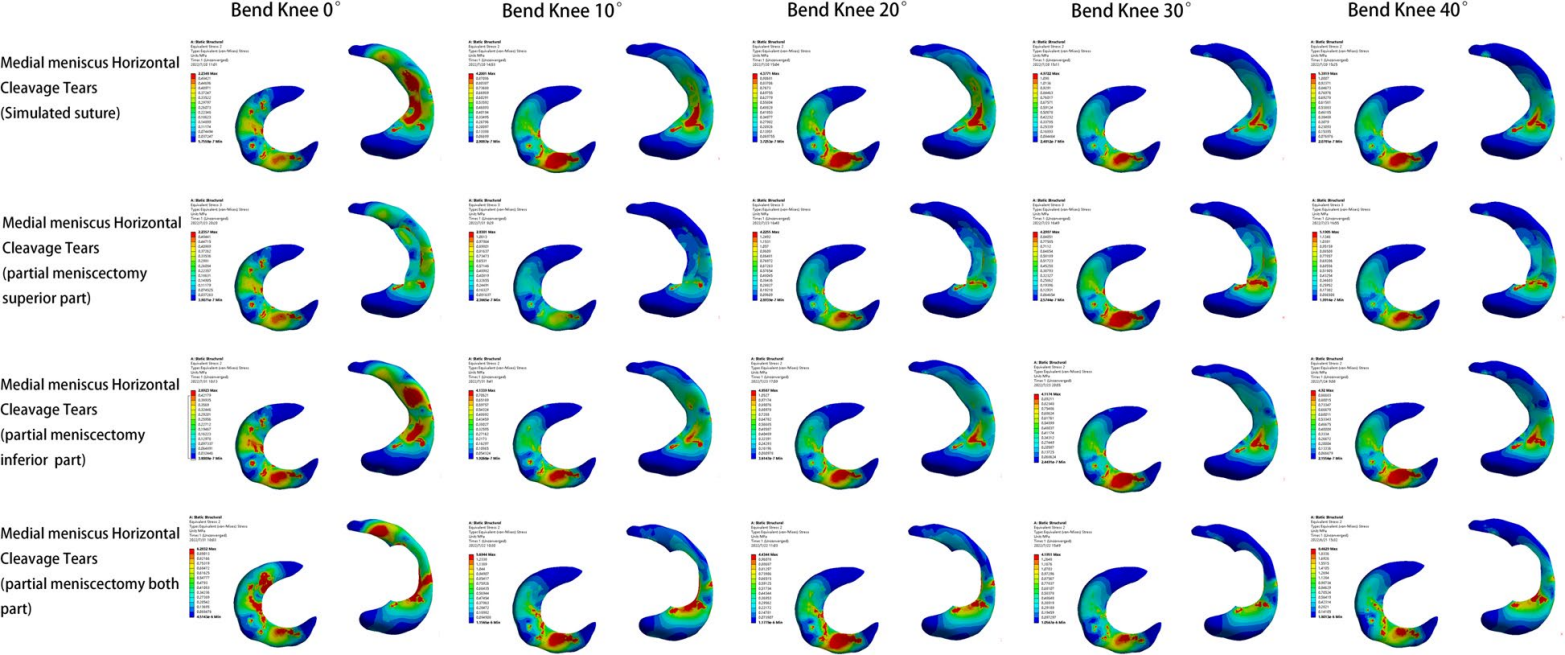
Meniscus pressure analysis at different angles in four medial meniscus injury models

**Fig. 7:**
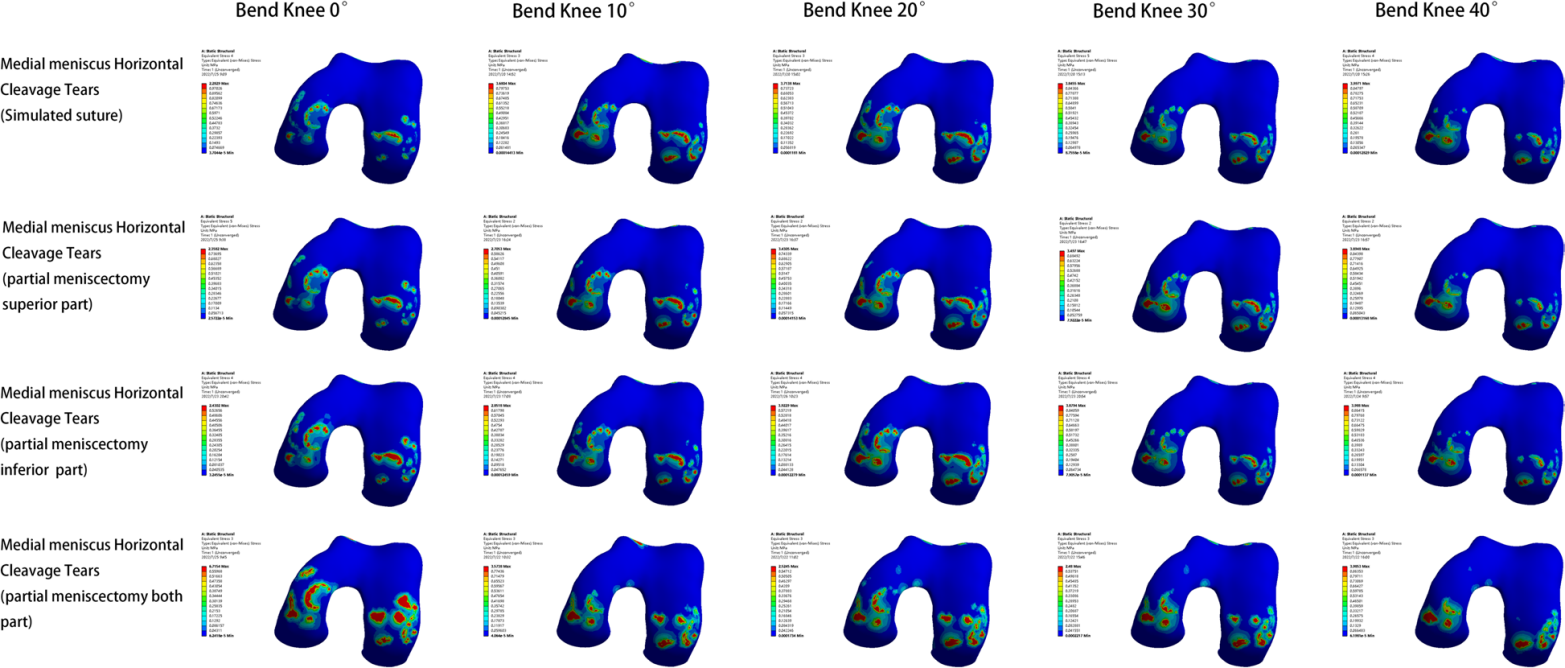
Analysis of femoral condyle cartilage pressure at different angles in four medial meniscus injury models

**Fig. 8:**
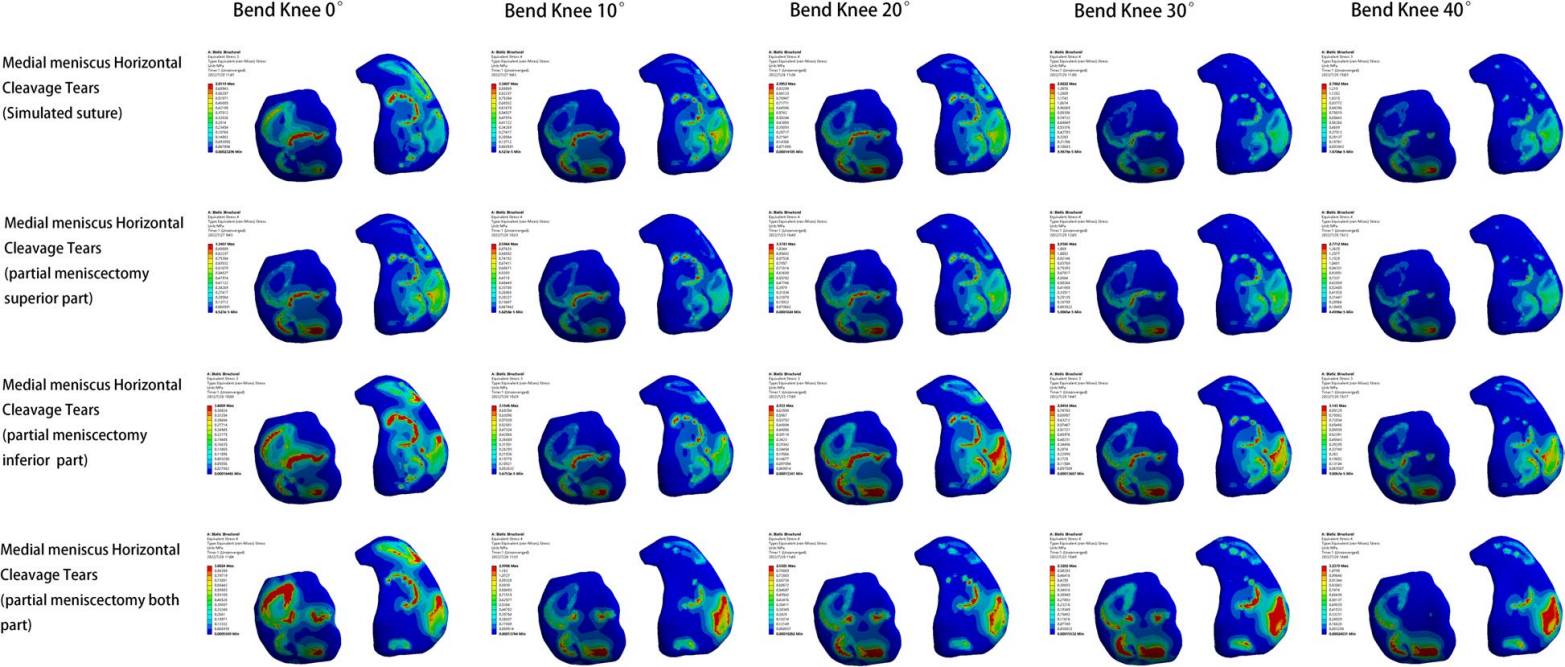
Analysis of tibial plateau cartilage pressure at different angles in four medial meniscus injury models

Within the four medial meniscus injury models, the lowest peak internal pressure of the knee joint was observed in the meniscal suture model, In contrast, the peak value of the bilateral lobulotomy model was larger. The pressure alterations in the superior leaflet resection model were most similar to those in the suture model. Knee flexion angle changes also impacted the pressure amounts. As the knee flexed from 0° to 40°, peak pressure increased, with a greater increase in the lateral compartment compared to the medial compartment. The primary stress is from the body of the meniscus on both sides to the body of the medial meniscus, the free edge, and the posterior corner of the lateral meniscus. The femoral condylar cartilage experienced a minor increase in peak pressure, with the largest increase occurring between 10° and 20°. The principal stress area gradually shifted from the anterior to the posterior of the weight-bearing zone. The main stress area steadily transitioned from the anterior and middle tibial plateau cartilage to the posterior plateau cartilage (Figs. 6, 7 and 8).

#### Models of lateral meniscus injury

**Fig. 9:**
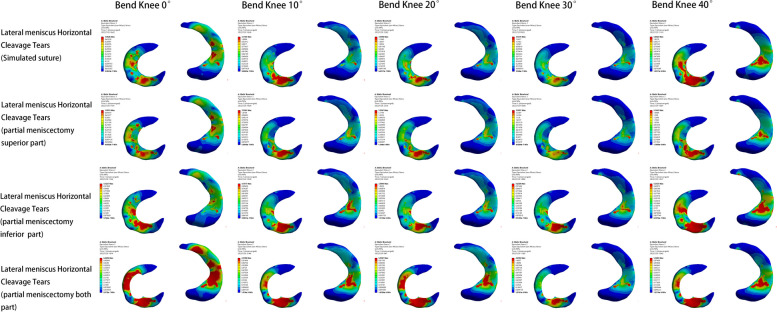
Meniscus pressure analysis at different angles in four models of lateral meniscus injury

**Fig. 10:**
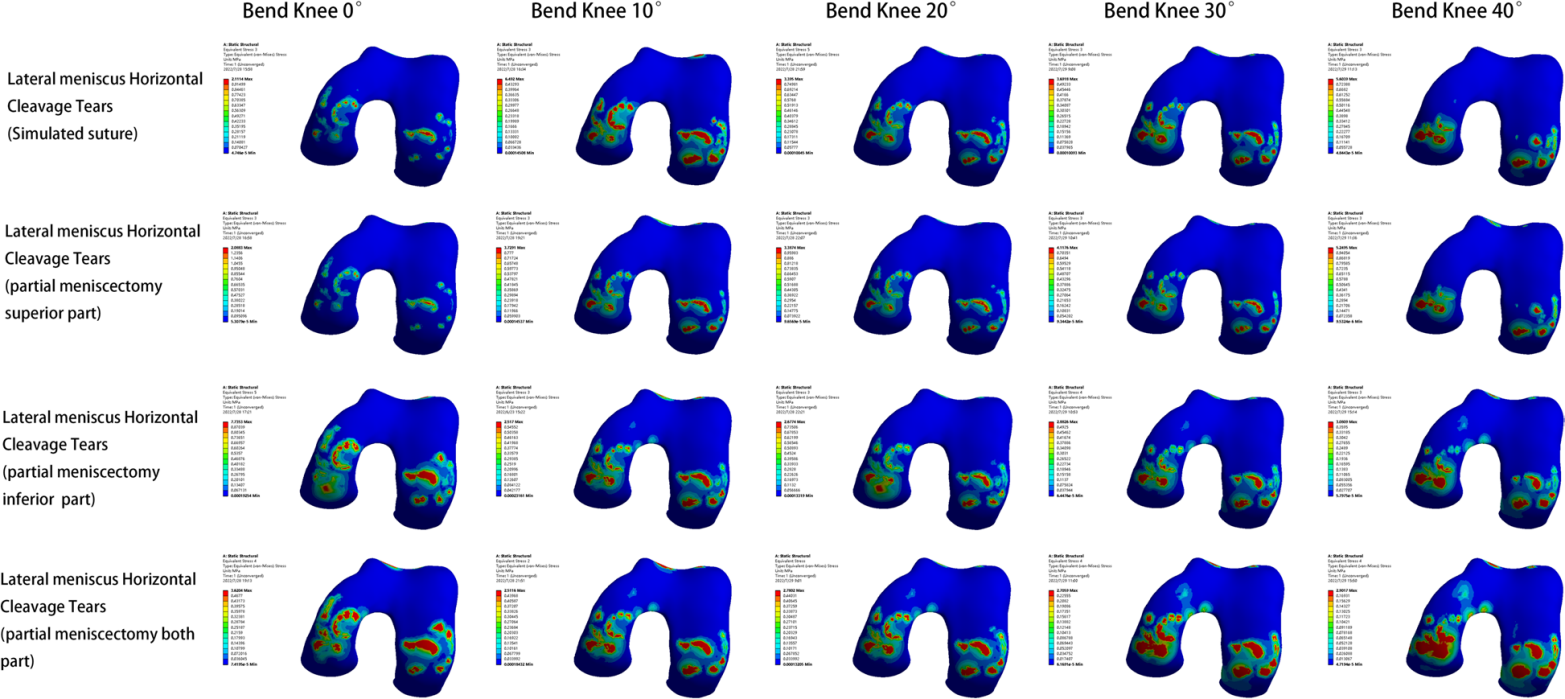
Analysis of femoral condyle cartilage pressure at different angles in four models of lateral meniscus injury

**Fig. 11:**
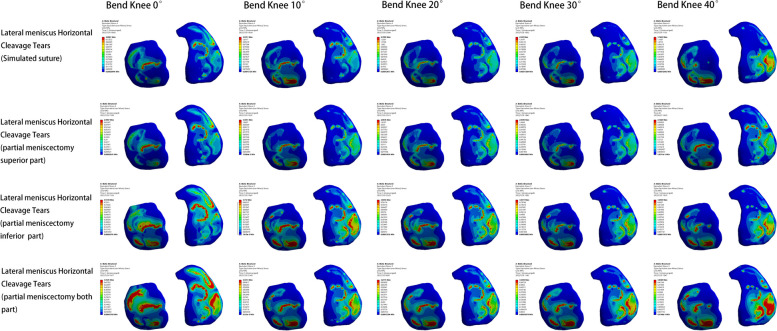
Analysis of tibial plateau cartilage pressure at different angles in four models of lateral meniscus injury

In the model of lateral meniscus injury, the lowest peak internal pressure of the meniscus, femoral condylar cartilage, and tibial plateau cartilage also appeared in the post-meniscal suture model. Except for the maximum pressure peak value of the meniscus in the bilateral leaflet resection model, the maximum pressure peak value of femoral condylar cartilage and tibial plateau cartilage appeared in the inferior leaflet resection model. The pressure changes during knee flexion also varied from the medial meniscus injury model: as the knee flexed from 0° to 40°, the peak pressure on the meniscus continuously increased, with the largest increase between 0° and 10°; the peak pressure on the femoral condylar cartilage initially increased and then gradually decreased, with the highest increase between 0° and 20°; the peak pressure on the tibial plateau cartilage first decreased gradually and then rose again, reaching an elevated level at 40° of knee flexion (Figs. 9, 10 and 11).

### Analysis of the internal shear force (Tresca stress) situation at different angles of knee flexion

#### Medial meniscus injury model

The lowest value of peak shear force (Tresca stress) within the knee joint was observed in the model following the meniscal suture, while the highest value was found in the bilateral leaflet resection model, exhibiting an increase of over 100%. The increase in shear force for the model after superior leaflet resection was less than 10%, and the increase in shear force for the model after inferior leaflet resection exceeded 50%. During knee joint flexion from 0° to 40°, the peak shear force progressively increased. Throughout knee flexion from 0° to 40°, the peak shear force initially decreased and subsequently increased again, with the largest increase in shear force occurring between 30° and 40°.

#### Lateral meniscus injury model

The minimum value of peak shear force within the knee joint was identified in the model following the meniscal suture. The maximum value emerged in the bilateral leaflet resection model, demonstrating an increase of 80%. The increase in shear force for the superior leaflet resection model was merely 4%, and the increase in shear force for the inferior leaflet resection model reached 40%. During knee flexion from 0° to 40°, the peak shear force on the meniscus and femoral condyle cartilage decreased and then increased, while the peak shear force on the tibial plateau cartilage also diminished and subsequently rose. The tibial plateau cartilage experienced a peak shear force that steadily increased before declining.

**Fig. 12:**
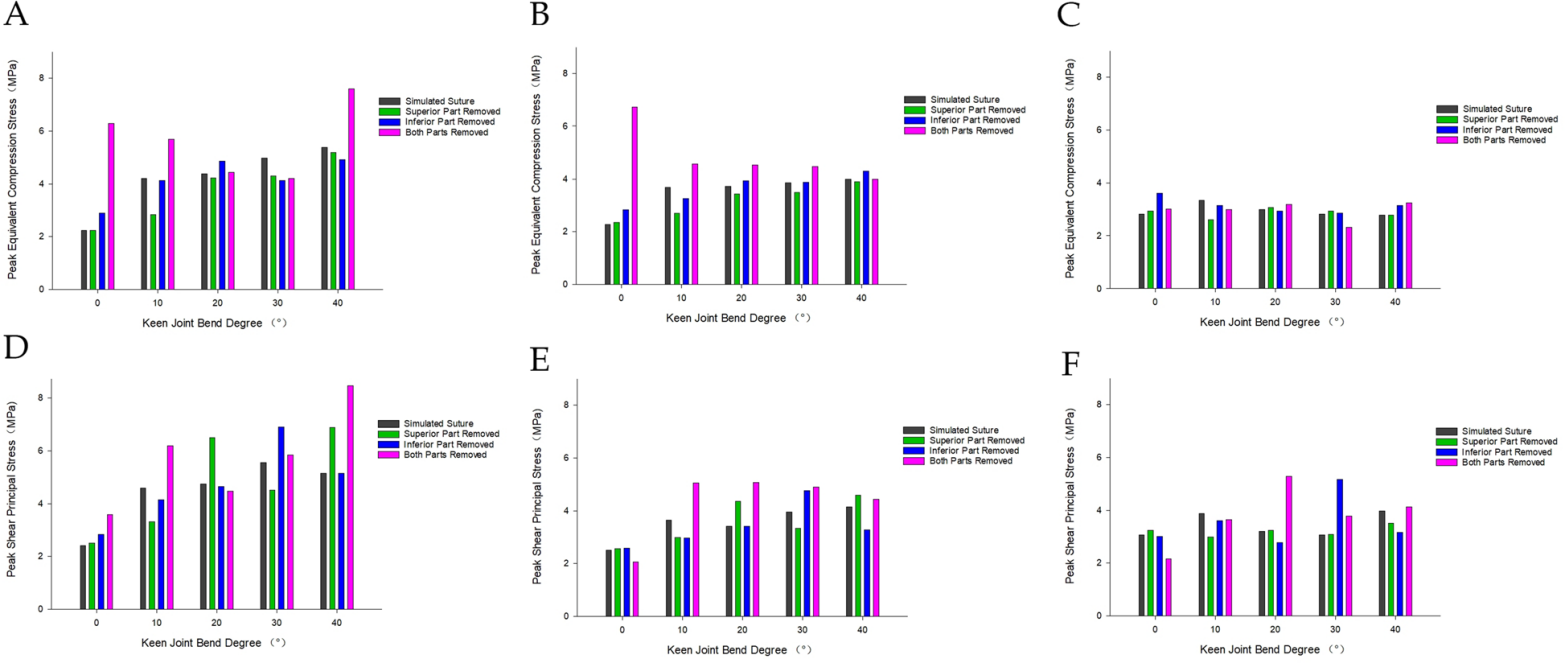
The trend of internal pressure/shear force in the knee joint model with medial meniscus injury. **A** Meniscus pressure change trend. **B** Femoral condyle cartilage pressure change trend. **C** Tibial plateau cartilage pressure change trend. **D** Trend of shear force on the meniscus. **E** Trend of shear force on femoral condyle cartilage. **F** Trend of shear force on tibial plateau cartilage

**Fig. 13:**
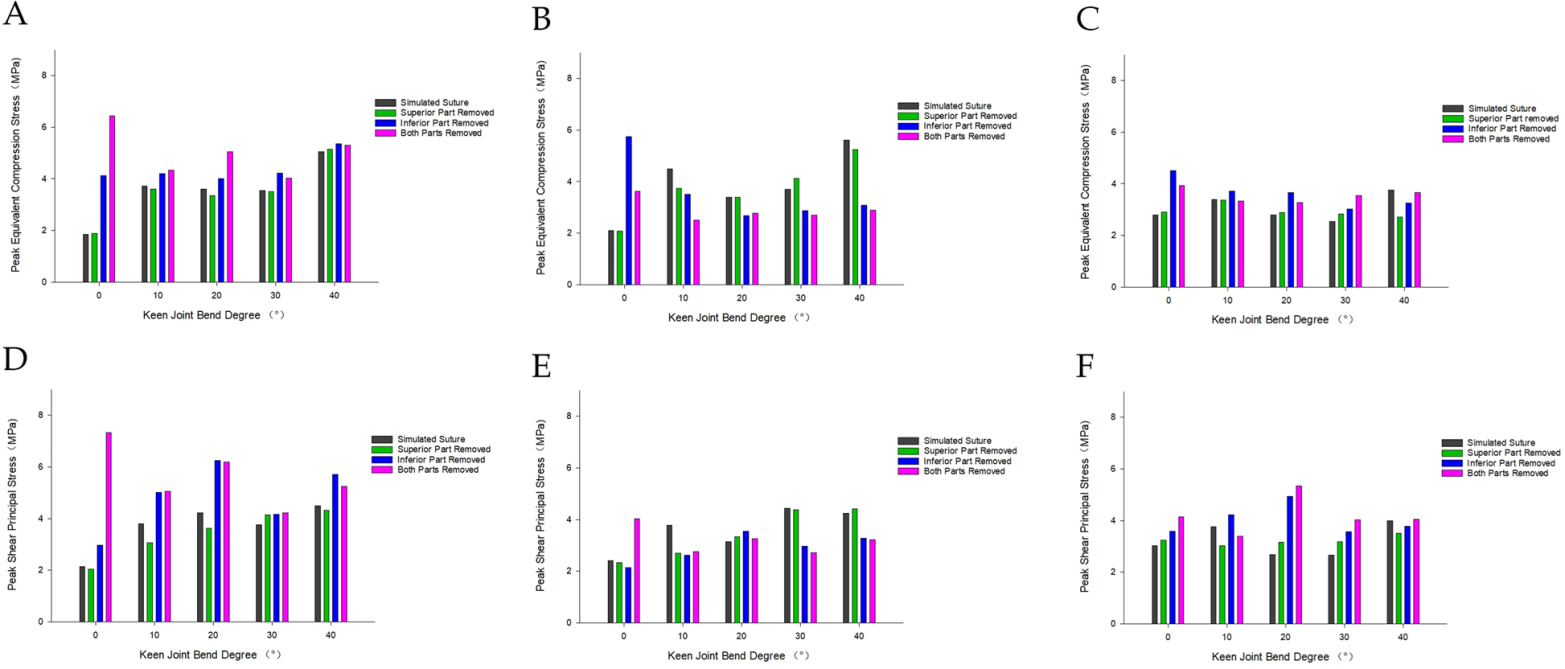
Change trend of internal pressure/shear force in the knee joint model with lateral meniscus injury. **A** Meniscus pressure change trend. **B** Femoral condyle cartilage pressure change trend. **C** Tibial plateau cartilage pressure change trend. **D** Trend of shear force on the meniscus. **E** Trend of shear force on femoral condyle cartilage. **F** Trend of shear force on tibial plateau cartilage

The patterns of internal pressure and shear force within the knee joint for both the medial meniscus injury model and the lateral meniscus injury model revealed that the overall shear force level was marginally higher than the pressure level. Additionally, the patterns of internal pressure and shear force in the knee joint following the resection of the superior leaflet of the horizontal meniscal fissure more closely resembled the patterns of internal pressure and shear force in the knee joint after meniscal suture (Figs. 12 and 13).

#### Analysis of the internal force area of the knee joint

**Fig. 14:**
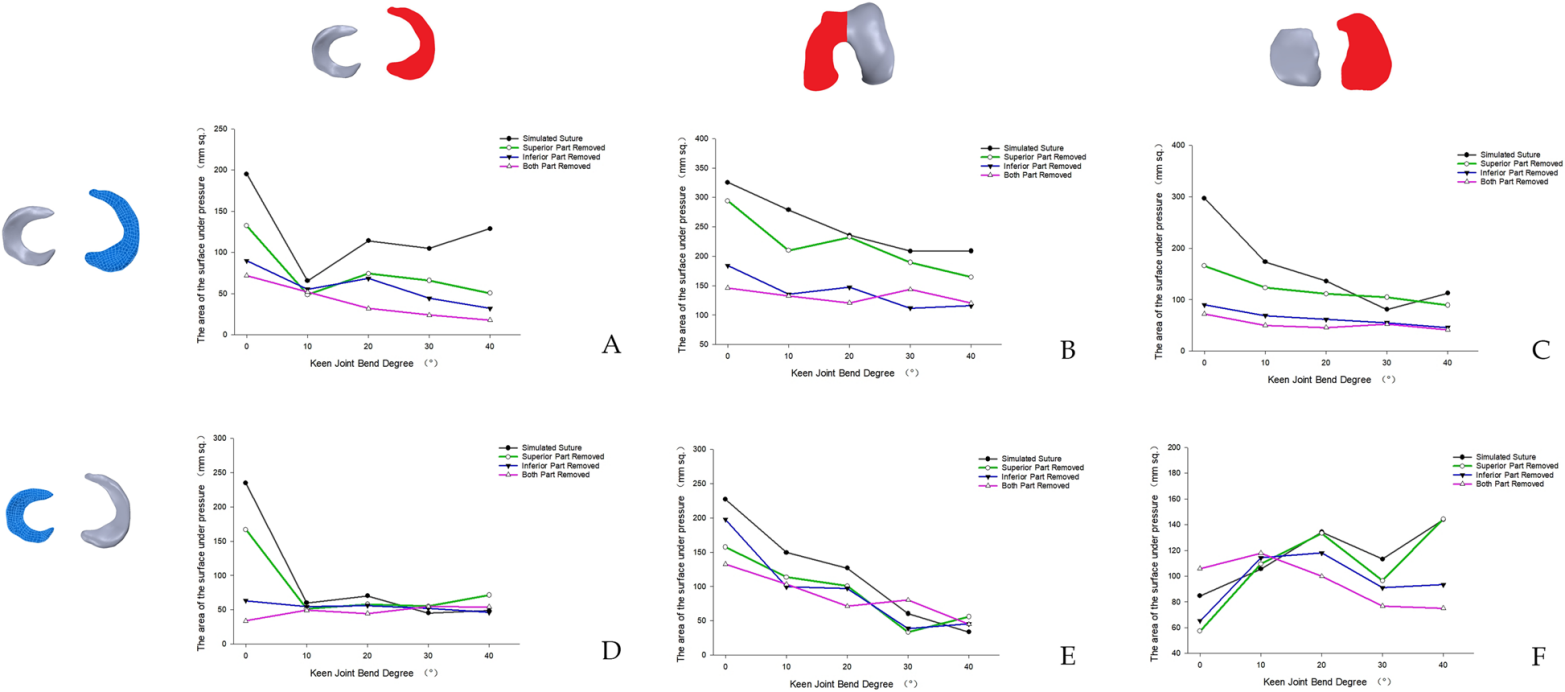
Force area of the medial compartment of the knee joint. **A** Variation trend of the force area of the medial meniscus in the medial meniscus injury model under different flexion angles. **B** Variation trend of the force area of the medial femoral condyle in the medial meniscus injury model under different flexion angles. **C** Variation trend of the force area of the medial tibial plateau in the medial meniscus injury model under different flexion angles. **D** Variation trend of the force area of medial meniscus in the model of lateral meniscus injury under different flexion angles. **E** Variation trend of the force area of the medial femoral condyle in the model of lateral meniscus injury under different flexion angles. **F** Variation trend of the force area of the medial tibial plateau in the model of lateral meniscus injury under different flexion angles

**Fig. 15:**
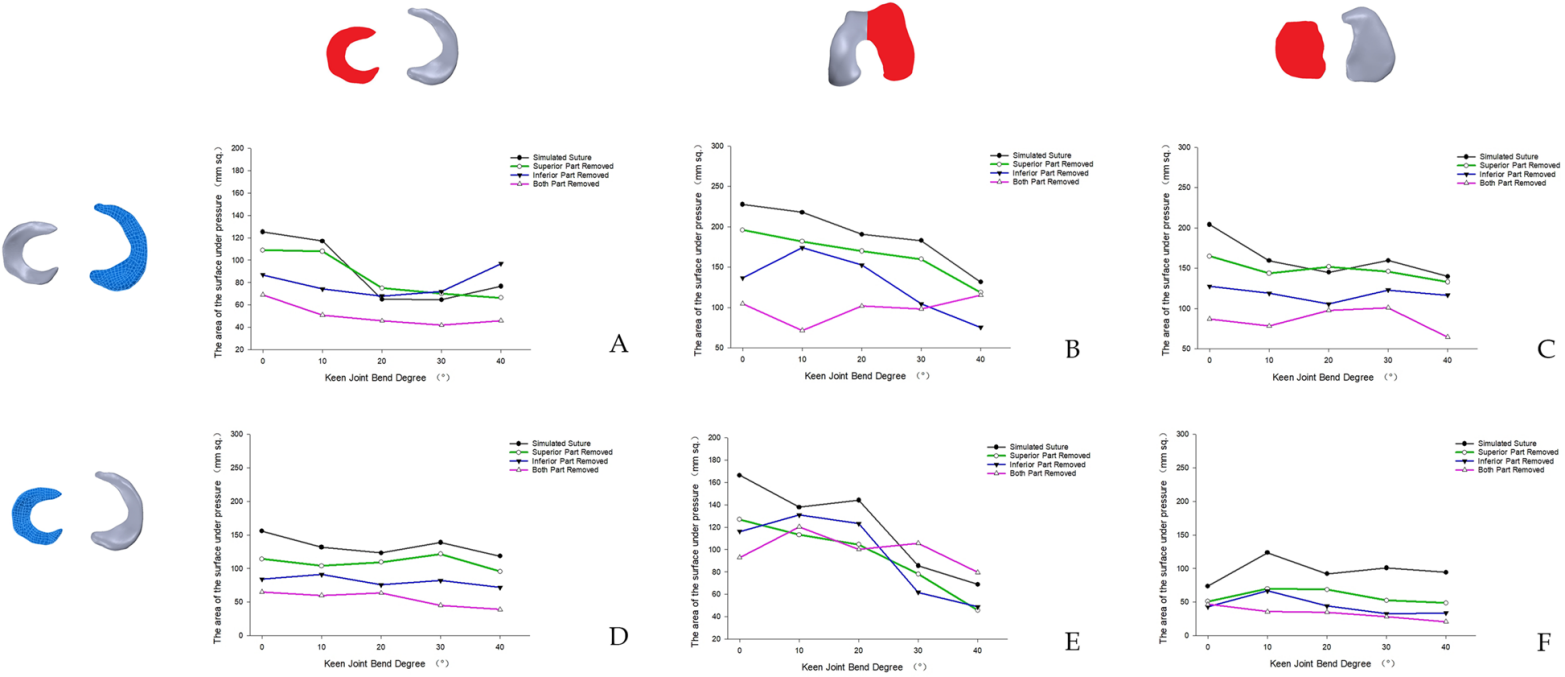
Force area of the lateral compartment of the knee joint (black: meniscus suture model; green: upper valvular lobectomy model; Blue: Lower valvular lobectomy model; Purple: double valvular lobectomy model). **A** Variation trend of the force area of the lateral meniscus in the medial meniscus injury model under different flexion angles. **B** Variation trend of the force area of the lateral femoral condyle in the medial meniscus injury model under different flexion angles. **C** Variation trend of the force area of the lateral tibial plateau in the medial meniscus injury model under different flexion angles. **D** Variation trend of the force area of the lateral meniscus in the model with different flexion angles. **E** Variation trend of the force area of the lateral femoral condyle in the model with different flexion angles. **F** Variation trend of the force area of the lateral tibial plateau in the model with different flexion angles

We found that after calculating the force areas of meniscus, femoral condyle and tibial plateau at 0°, 10°, 20°, 30° and 40° in different models by Image J software, the maximum force area inside the knee joint was simulated suture meniscus model in both the total knee model after medial meniscus injury and the total knee model after lateral meniscus injury, in the medial meniscus injury model, the maximum area of force on the medial compartment was 517.31 mm^2^ and the maximum area of force on the lateral compartment was 557.02 mm^2^, which appeared in the simulated post-suture model at 0° of knee flexion; the minimum area of force on the medial compartment was 175.63 mm^2^ and the minimum area of force on the lateral compartment was 226.01 mm^2^, which appeared in the bilateral leaflet resection model of the medial meniscus at 40° of knee flexion; in the lateral In the lateral meniscus injury model, the maximum force area of the medial interventricular was 546.25 mm^2^ and the maximum force area of the lateral interventricular was 595.62 mm^2^, which occurred in the simulated post-suture model at 0° of flexion; the minimum force area of the medial interventricular was 174.06 mm^2^ and the minimum force area of the lateral interventricular was 139.7 mm^2^, which occurred in the medial meniscus bilobar resection model at 40° of flexion model after resection. We also found that the internal force area of the knee decreased regardless of the resection method, and the internal force area of the knee after superior meniscal leaflet resection was the closest to the internal force area of the knee after the meniscal suture (Figs. 14 and 15).

## Discussion

It is currently accepted that the optimal treatment option for small horizontal meniscal tears is arthroscopic repair of the torn meniscus using a suture system, which not only improves the probability of healing of the meniscus but also preserves the internal cushion of the knee joint to the greatest extent possible [32–34], but because the repair of horizontal tears is more difficult to perform microscopically than longitudinal tears, and because some studies have shown that patients repaired by sutures have a higher complication rate are higher in patients who underwent partial resection [35], so most physicians prefer partial resection rather than attempting repair when dealing with horizontal meniscal tears [36–38]. However, when we remove a portion of the meniscus, it inevitably results in a reduction of the contact area within the knee joint, an increase in the peak pressure within the intercompartment, and ultimately causes cartilage wear and tear thereby increasing the risk of osteoarthritis [7, 39, 40]. If a partial meniscectomy with postoperative results close to those of suture repair could be found, it might eliminate clinicians’ hesitation when faced with horizontal meniscal tears.

In the present study, we established a complete knee model by computer simulation and simulated the effect after a meniscal horizontal tear and three meniscectomy procedures, and then loaded loads for mechanical analysis separately to obtain data on the pressure, shear force, and force area on the meniscus, femoral condyle cartilage, and tibial plateau cartilage. It has been found through cadaveric studies that the contact area within the knee joint does not change when the meniscus has a horizontal tear when the inferior leaflet is removed alone [41], and we found experimentally that the force area on the meniscus, femoral condyle cartilage, and tibial plateau cartilage decreased regardless of which leaflet was removed, with the greatest reduction in area after double leaflet removal, which is consistent with Beamer’s [3] results. We also found that the change in pressure or shear force within the knee joint after resection of the superior leaflet alone compared with the resection of the inferior leaflet was closest to the change in pressure after the horizontal meniscal fracture suture, which is different from our previous perception. This may occur because the superior meniscal leaflet matches the shape of the femoral condyle, forming a curved concave surface, whereas the inferior leaflet matches the tibial plateau, which is closer to a flat surface. After the removal of the superior leaflet, the meniscal contact area can be more compensated by force deformation, whereas the deformation after the removal of the inferior leaflet can only lose more matching, leading to a decrease in the contact area and an increase in peak pressure. Previously, our principle for removal of meniscal leaflets was to remove the unstable leaflet first, and if both leaflets were stable, then removal of the inferior leaflet was preferred, but our experimental results do not seem to support that removal of the inferior leaflet while preserving the superior leaflet is an ideal choice.

The data reveal that the internal force area of the bilateral interval of the knee decreases with increasing flexion angle from 0° to 40°, which is consistent with the results of a cadaveric study by Morimoto [42]. Interestingly, in the medial meniscal injury model, the reduction in the force surface of the medial compartment is large, while the reduction in the force area of the lateral compartment is more modest, perhaps because the contact area lost in the medial compartment is partially shared to the lateral compartment in order to maintain knee stability, resulting in partial compensation for the reduction in the force area of the lateral compartment, but this situation seems to further exacerbate the lateral However, this appears to further exacerbate the degeneration of the lateral interventricular compartment, yet we did not find significant medial–lateral interventricular compensation in the lateral meniscal injury model.

The limitation of the current study is that the effect of various treatments of horizontal meniscal tears on the internal pressure and shear forces of the knee joint was only analyzed by computer simulation, lacking biomechanical data performed on cadavers and supported by relevant clinical trial data.

In summary, we simulated four different management modalities for different horizontal meniscal tears with a complete three-dimensional knee model and analyzed the changes in pressure, shear force, and force area inside the knee joint under different flexion angles.

## Conclusion

Our experimental results show that suture repair is certainly the best way to maintain the internal force relationship of the knee joint, however, in cases where suture repair is difficult, the option of removing the superior meniscal leaflet is also a more reliable option.

## Data Availability

The datasets generated and/or analysed during the current study are not publicly available due privacy of the patient but are available from the corresponding author on reasonable request.
